# Management of Trastuzumab Deruxtecan-related nausea and vomiting in real-world practice

**DOI:** 10.3389/fonc.2024.1374547

**Published:** 2024-03-11

**Authors:** Giulia Notini, Matteo Maria Naldini, Lorenzo Sica, Giulia Viale, Alessia Rognone, Stefania Zambelli, Patrizia Zucchinelli, Marta Piras, Carlo Bosi, Marco Mariani, Daniela Aldrighetti, Giampaolo Bianchini, Luca Licata

**Affiliations:** ^1^ Department of Medical Oncology, IRCCS San Raffaele Scientific Institute, Milan, Italy; ^2^ School of Medicine and Surgery, Vita-Salute San Raffaele University, Milan, Italy

**Keywords:** nausea, vomiting, antiemetic prophylaxis, Trastuzumab Deruxtecan, breast cancer

## Abstract

**Background:**

Nausea and vomiting are common side effects of Trastuzumab Deruxtecan (T-DXd), but guidelines for optimal management were not initially available. This retrospective single-center study aimed at evaluating the efficacy of two antiemetic regimens in patients receiving T-DXd.

**Methods:**

Data from metastatic breast cancer patients receiving T-DXd were collected. Two groups were defined: patients treated with 5-HT3 receptor antagonists (RA) ± dexamethasone (5-HT3-group) and patients treated with a fixed oral combination of netupitant (NK1RA) and palonosetron ± dexamethasone (NK1 group). Physicians preferentially offered the NK1 regimen to patients at higher risk of nausea and vomiting based on internal recommendations. Only nausea and vomiting during cycles 1 and 2 were considered. Comparisons of nausea and vomiting by the antiemetic prophylaxis group were assessed using chi-square.

**Results:**

A total of 53 patients were included in the analysis. At cycle 1, 72% and 28% of patients received the 5-HT3 and NK1 prophylaxis, respectively. Overall, 58% reported nausea, with no differences between groups (58% vs. 60%; *p* = 0.832), but with a trend for lower grade in the NK1 group (33.3% G1; 26.7% G2) compared to the 5-HT3 group (23.7% G1; 31.6% G2; 2.6% G3). Vomiting was reported by 21% and 0% of patients in the 5-HT3 and the NK1 group, respectively (*p* = 0.054). Among the 15 patients in the 5-HT3 group with nausea at cycle 1 who escalated to NK1 at cycle 2, nausea decreased from 100% to 53% (*p* = 0.022) and vomiting decreased from 47% to 13% (*p* = 0.046).

**Conclusions:**

The NK1 regimen improved vomiting control at cycle 1 and, when introduced at cycle 2, significantly improved both nausea and vomiting. The biased NK1 selection for higher-risk patients may have dampened the differences between groups at cycle 1. These findings support enhanced control of T-DXd-related nausea and vomiting with NK1RA.

## Introduction

Trastuzumab Deruxtecan (T-DXd) is a HER2-directed antibody–drug conjugate (ADC) composed of a humanized immunoglobulin G1 anti-HER2 monoclonal antibody and a topoisomerase I inhibitor cytotoxic payload, covalently linked by a tetrapeptide-based cleavable linker (1).

T-DXd has demonstrated clinically meaningful activity across a broad range of HER2-expressing solid tumors (2) and is currently approved as a standard of care treatment for HER2-expressing metastatic breast cancer, HER2-positive gastric or gastroesophageal junction adenocarcinoma, and HER2-mutant non-small cell lung cancer (3–8).

Despite its generally acceptable safety profile, T-DXd presents some non-negligible side effects that require appropriate clinical management. Specifically, it has been linked to a notable occurrence of nausea and vomiting, reaching 75% and 45%, respectively, in clinical trials (3–8).

These side effects may arise very early in the treatment course and persist for an extended period (9), inevitably exerting a negative impact on the quality of life for patients if not adequately managed (10).

At the time of its initial clinical development, the emetogenic potential of T-DXd was not well known, and early trial protocols did not include recommendations for any specific antiemetic prophylaxis. Furthermore, guidelines for the optimal management of T-DXd-related nausea and vomiting were not available until 2020, when the ASCO and NCCN guidelines categorized T-DXd as a moderate emetic risk anticancer agent (11, 12). Therefore, the early management of T-DXd-related nausea and vomiting was mostly empirical.

Between November and December 2020, three virtual focus groups of Italian oncologists were held to raise awareness of the importance of appropriate antiemetic prophylaxis for T-DXd and to provide oncologists with practical guidance for effective management (13). Consistent with the 2020 ASCO and NCCN guidelines, the panelists endorsed the antiemetic protocol defined at the San Raffaele Hospital, recommending a two-drug prophylaxis regimen with a 5-HT3 receptor antagonist (RA) plus dexamethasone for most patients, with the option to add an NK1 RA from the first cycle in the presence of individual risk factors, or at subsequent cycles in case of suboptimal control of nausea during cycle 1 (13).

Here, we present real-world data regarding the management of T-DXd-related nausea and vomiting in patients with metastatic breast cancer using this antiemetic protocol.

## Materials and methods

### Patients

Data from patients with metastatic breast cancer who had received T-DXd as of February 2023 were retrospectively collected from the healthcare services’ information system of the San Raffaele Hospital in Milan, Italy. The available information on the antiemetic prophylaxis used and the occurrence of nausea and vomiting at cycle 1 and subsequent cycles of T-DXd treatment were recorded. Only patients with reliable information on antiemetic prophylaxis type and severity of nausea and vomiting according to the Common Terminology Criteria for Adverse Events (CTCAE) version 5.0 were included in the analysis.

### Procedures

Two groups of patients were defined: those treated with a 5-HT3 RA with or without dexamethasone (5-HT3 group) and those treated with a fixed oral combination of NK1RA (netupitant) and palonosetron with or without dexamethasone (NK1 group). Patients considered at higher risk of nausea and vomiting by physicians according to their individual risk factors were preferentially offered the NK1RA-containing regimen in line with internal recommendations (13). Only nausea and vomiting occurring at cycles 1 and 2 were considered for the analysis. Grading of nausea and vomiting was assessed by the treating physician and recorded in the medical chart.

### Statistics

Descriptive statistics were used to summarize patient and tumor characteristics. Comparisons of nausea and vomiting by the antiemetic prophylaxis group were assessed using Pearson’s chi-squared test. All *p*-values were two-sided, and statistical significance was set at *p* ≤ 0.05. Only univariate analysis was performed.

### Ethical issues

This retrospective study analyzed data that have been anonymized from previously collected patient information and did not involve any intervention or impact on patient care. All patients had provided informed consent before undergoing treatment with T-DXd. The study protocol (“TD-rNV”) was approved by the institutional Ethics Committee and followed local regulation and ethical guidelines.

## Results

Between July 2018 and February 2023, 62 patients with HER2+ metastatic breast cancer were treated with T-DXd at our institution. After excluding patients without reliable information on antiemetic prophylaxis type and severity of nausea and vomiting, 53 patients were included in the analysis.

Baseline patient and disease characteristics are reported in **Table 1**. Median age was 56 years (range, 24–85 years); 75% of patients had received two or more previous lines of therapy for metastatic disease; 57% had received TDM1 as the latest systemic therapy. Thirty-six percent of patients had liver metastases and 26% had a history of brain metastases.

**Table 1:** Patient and disease baseline characteristics.

Characteristics	Overall (*N* = 53)	5-HT3 group (*N* = 38)	NK1 group (*N* = 15)
Age			
**Range**	24–85	24–85	46–80
**Median**	56	52	60
**ECOG PS**			
**0**	42	30	12
**1**	11	8	3
No. of prior lines for metastatic disease			
**Range**	0-12	0-5	1-12
**0**	3	3	0
**1**	10	8	2
**2**	26	21	5
**≥3**	14	6	8
Last systemic therapy			
**TDM1**	30	23	7
**Trastuzumab or lapatinib ± HT**	12	8	4
**CT ± trastuzumab or lapatinib**	9	5	4
**HT ± CDK4/6i**	2	2	0
Liver metastases			
**No**	34	28	6
**Yes**	19	10	9
History of brain metastases			
**No**	39	25	14
**Yes**	14	13	1

ECOG PS, Eastern Cooperative Oncology Group performance status; HT, hormone therapy; CT, chemotherapy; NK1RA, neurokinine 1 receptor antagonist.

At cycle 1, 28% and 72% of patients received antiemetic prophylaxis with a three-drug (NK1 group) and a two-drug (5-HT3 group) regimen, respectively.

**Figure 1:**
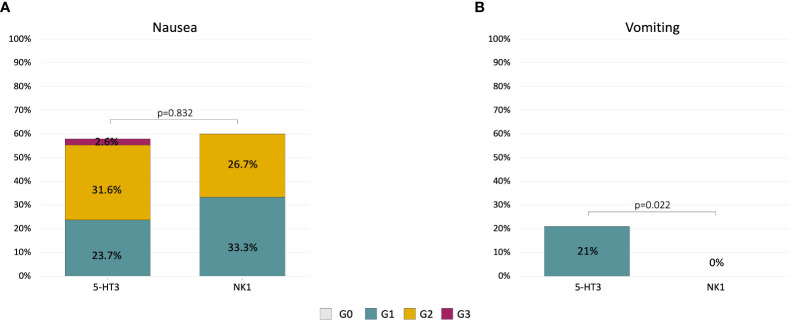
Rates of nausea **(A)** and vomiting **(B)** at cycle 1 in the 5-HT3 and NK1 group, respectively.

At cycle 1, 58% of patients reported nausea of any grade, with no significant differences between groups (58% in the 5-HT3 group and 60.0% in the NK1 group, *p* = 0.832) (**Figure 1A**). A numerical trend for lower-grade nausea in the NK1 group compared to the 5-HT3 group was observed. Nausea of grades 1, 2, and 3 was recorded in 24%, 32%, and 3%, respectively, in the 5-HT3 group, compared to 33%, 27%, and 0%, respectively, in the NK1 group (**Figure 1A**).

Overall, 15% of patients reported vomiting of any grade during cycle 1, 21% in the 5-HT3 group (all G1) and 0% in the NK1 group (*p* = 0.054) (**Figure 1B**).

**Figure 2:**
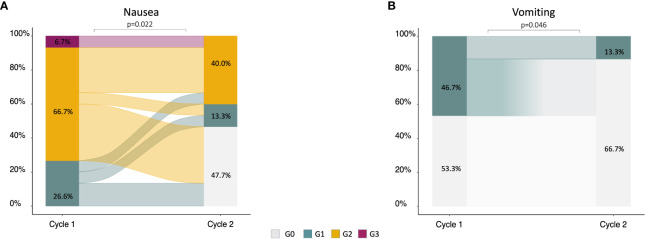
Alluvial plots describing the change of nausea **(A)** and vomiting **(B)** severity in patients (*n* = 15) who did not achieve an optimal control of symptoms at cycle 1 with 5-HT3 and escalated to the NK1 regimen at cycle 2.

Among the 22 patients in the 5-HT3 group with nausea at cycle 1, 15 patients immediately escalated to the NK1 regimen at cycle 2. Within this group, a statistically significant reduction of nausea and vomiting incidence was observed at cycle 2. Nausea decreased from 100% any grade (26% G1, 67% G2, and 7% G3) at cycle 1 to 53% any grade (13% G1, 40% G2) at cycle 2 (*p* = 0.022) (**Figure 2A**). Vomiting decreased from 47% (all G1) at cycle 1 to 13% (all G1) at cycle 2 (*p* = 0.046) (**Figure 2B**).

No dose reductions or interruptions have been observed in either group during the first two cycles.

## Discussion

Nausea and vomiting are among the most common toxicities associated with T-DXd. Since these side effects can significantly impair patient’s quality of life and adherence to treatment, their proper management is essential to ensure that patients derive the maximum benefit from T-DXd, while avoiding potential detrimental consequences.

Based on early clinical trial data, T-DXd was previously categorized as moderately emetogenic (11, 12). However, with the maturation of real-world experience suggesting an inadequate nausea control with the two-drug prophylaxis in a non-negligible proportion of patients (13, 14), in January 2023, the NCCN guidelines re-categorized T-DXd as a highly emetogenic agent, modifying the recommendation to endorse a three-drug antiemetic regimen for all patients (15). In January 2024, the ESMO guidelines have incorporated explicit recommendations for the management of T-DXd-related nausea and vomiting, categorizing it as a pharmaceutical agent at the high end of the moderate category (16). The recommendations advise the implementation of a three-drug regimen that includes an NK1RA, aligning with the established approach for Carboplatin with AUC ≥5 (16).

Recently, a randomized study comparing antiemetic prophylaxis with either a two- or three-drug regimen for patients with breast cancer scheduled to receive T-DXd was conducted (17). Patients were randomly assigned to receive granisetron and dexamethasone or granisetron, dexamethasone, and aprepitant. The primary endpoint was complete response rate, defined as no emesis or no rescue therapy during the overall phase (0–120 h after initiating T-DXd). Complete response rates were 36.8% and 70.0% with the two- and the three-drug regimen, respectively (odds ratio = 0.1334; *p*-value = 0.019), with a more pronounced difference between the two regimens in the delayed phase (24–120 h, 36.8% vs. 75%) and extended-delayed phase (24–168 h, 31.6% vs. 70%) (17).

Our real-world experience aligns with these data and further emphasizes that a two-drug antiemetic prophylaxis cannot be considered effective. The NK1RA-based regimen demonstrated a meaningful improvement in vomiting control at cycle 1 and, when introduced as rescue at cycle 2, significantly improved both nausea and vomiting. The biased preferential selection of the NK1 regimen for higher-risk patients may have dampened the differences between the two- and the three-drug regimen in nausea control at cycle 1 and therefore an improved control of nausea with the NK1 regimen in an unselected population cannot be ruled out.

Nevertheless, these data indicate that despite the use of a three-drug prophylaxis regimen including an NK1RA, some patients may continue to experience nausea and vomiting during T-DXd treatment. This could be at least partly attributed to the unique pharmacokinetic properties of T-DXd. Indeed, pharmacokinetic studies showed that free molecules of exatecan, the T-DXd payload, are released into the systemic circulation at levels that remain pharmacologically active for extended durations (18, 19), supporting the hypothesis that circulating free payloads could contribute not only to the efficacy but also to the toxicity profile of T-DXd. This phenomenon may lead to the activation of different pathways of nausea and vomiting, potentially necessitating antiemetic strategies that remain effective for an extended period.

Olanzapine is an atypical antipsychotic drug with broad-spectrum antiemetic properties, attributed to its action on multiple neurotransmitter receptors (20, 21). Several studies demonstrated that olanzapine-containing three- or four-drug antiemetic regimens are effective for preventing acute and delayed nausea and vomiting with both moderately or highly emetogenic anticancer regimens (22, 23). Moreover, some data suggest that, when combined with 5-HT3 RA and dexamethasone, olanzapine is more effective than an NK1 RA in preventing delayed nausea (24, 25). For this reason, there is an increasing interest in investigating the use of olanzapine to prevent and treat nausea and vomiting linked to T-DXd. The ongoing ERICA study (26) is a multicenter, randomized, double-blind, placebo-controlled phase II trial designed to assess the effectiveness of an olanzapine-based antiemetic regimen for the management of T-DXd-related nausea and vomiting in patients with HER2-positive metastatic breast cancer. Although the results of this study will certainly provide a contribution to the field, it is noteworthy that the study was initiated when T-DXd was still classified as moderately emetogenic. Therefore, some may raise concerns about the appropriateness of the control arm, represented by the two-drug regimen of 5HT3 RA and dexamethasone.

Despite the demonstrated cost-effectiveness of olanzapine (27), clinicians sometimes hesitate to prescribe it due to concerns related to its toxicity profile, which could, in turn, impact patients’ quality of life. In fact, common adverse events with olanzapine include fatigue, postural hypotension, anticholinergic side effects, and sedation (28). Increasing evidence suggests that a low dose of olanzapine (i.e., 5 mg PO daily) may be equally effective but better tolerated than the initial standard 10-mg dose (29, 30), and even lower doses (2.5 mg daily) may provide a comparable antiemetic effect with increased patient tolerability (31).

Low-dose olanzapine may represent an additional useful agent for the management of T-DXd-related nausea and vomiting, particularly in those cases with a delayed presentation of symptoms.

Strengths of our study include its substantial sample size, as to the best of our knowledge, it represents the largest real-world series investigating T-DXd-related nausea and vomiting to date. Furthermore, the monocentric nature of this study allows for high homogeneity in the selection of antiemetic prophylaxis, adding to the reliability of our findings. However, there are limitations to our analysis. Firstly, because of the retrospective nature of the study, it was not possible to control for the distribution of all variables that could have influenced the results, such as concomitant medications that might interfere with the onset of nausea and vomiting during treatment or individual patient risk factors for chemotherapy-induced nausea and vomiting, such as history of alcohol intake, morning sickness, motion sickness, anxiety, and history of vomiting during prior therapy. Secondly, some patients did not receive dexamethasone within the antiemetic prophylaxis because they initiated T-DXd treatment in 2018, a period during which concerns about potential interactions between dexamethasone and T-DXd existed. This may have partially dampened the overall efficacy of prophylaxis. Moreover, for this analysis, we did not consider data regarding symptomatology occurring beyond cycle 2, and therefore, we did not collect late events that might have led to treatment delays or dose reductions in subsequent cycles. Nevertheless, it is well known that T-DXd-related nausea occurs at the beginning of treatment, and the likelihood of these events occurring for the first time after cycles 2 or 3 is extremely low (9). Lastly, the monocentric nature of the study may also be considered a drawback and could potentially limit the generalizability of the observed results.

In conclusion, the effective management of T-DXd-related nausea and vomiting is critical to enhance patients’ quality of life and ensure optimal treatment adherence. While common, these symptoms can be effectively managed in the majority of cases with the use of adequate antiemetic prophylaxis protocols. Our findings support enhanced control of nausea and vomiting with the use of a three-drug antiemetic regimen including an NK1RA, in line with the most recent antiemetic guidelines for T-DXd.

## Data availability statement

The raw data supporting the conclusions of this article will be made available by the authors, without undue reservation.

## Ethics statement

The studies involving humans were approved by San Raffaele Hospital institutional review board. The studies were conducted in accordance with the local legislation and institutional requirements. This retrospective study analyzed data that have been anonymized from previously collected patient information and did not involve any intervention or impact on patient care. All patients had provided informed consent before undergoing treatment with T-DXd.

## Author contributions

GN: Conceptualization, Data curation, Investigation, Methodology, Writing – original draft, Writing – review & editing. MN: Writing – original draft, Writing – review & editing. LS: Writing – original draft, Writing – review & editing. GV: Writing – original draft, Writing – review & editing. AR: Writing – original draft, Writing – review & editing. SZ: Writing – original draft, Writing – review & editing. PZ: Writing – original draft, Writing – review & editing. MP: Writing – original draft, Writing – review & editing. CB: Writing – original draft, Writing – review & editing. MM: Writing – original draft, Writing – review & editing. DA: Writing – original draft, Writing – review & editing. GB: Conceptualization, Funding acquisition, Supervision, Writing – original draft, Writing – review & editing. LL: Conceptualization, Data curation, Formal Analysis, Methodology, Supervision, Validation, Writing – original draft, Writing – review & editing.
